# YiQi YangYin Decoction Attenuates Nonalcoholic Fatty Liver Disease in Type 2 Diabetes Rats

**DOI:** 10.1155/2021/5511019

**Published:** 2021-09-28

**Authors:** Fengjin Li, Genli Liu, Piliang Xue, Zhen Ren, Peifang Dai, Wenying Niu, Mu Xin

**Affiliations:** ^1^The Heilongjiang University of Chinese Medicine, Harbin 150040, China; ^2^Heilongjiang Academy of Traditional Chinese Medicine, Harbin 150036, China

## Abstract

**Background:**

YiQi YangYin Decoction (YQ) is a modern Chinese formula composed by the guidance of traditional Chinese medicine theory, which consists of nine traditional Chinese medicines and is applied to treat type 2 diabetes mellitus (T2DM) with nonalcoholic fatty liver in clinic in China for more than a decade. This study aims to evaluate the antidiabetes and lipid-lowering effect of YQ and explore the possible mechanisms of this action.

**Methods:**

T2DM rat models were established and given YQ at three different doses for three weeks. Tissues, including pancreas islet and liver, and blood serum were collected. The levels of fasting blood glucose (FBG), fasting insulin (Fins), lipid index, such as total cholesterol (TC), triglyceride (TG), high-density lipoprotein (HDL), and low-density lipoprotein (LDL), and hepatic function index such as alanine aminotransferase (ALT), aspartate aminotransferase (AST), and alkaline phosphatase (ALP) in serum were measured. Pancreas islet damage and liver damage were observed by hematoxylin and eosin staining. The glycogen content and lipid accumulation in liver were determined by periodic acid-Schiff (PAS) staining and Oil Red O staining. The expression levels of insulin receptor substrate 2 (IRS-2), phosphatidylinositol 3 kinase-associated *p*85alpha (PI3K *p*85*α*), AKT, and Glucose Transporter 2 (Glut4) in pancreas islet and AMP-activated protein kinase alpha (AMPK*α*), sterol regulatory element-binding protein 1c (SREBP1c), acetyl-CoA carboxylase (ACC1), and peroxisome proliferator‐activated receptor-*α* (PPAR*α*) in liver were determined by western blotting. The relative expressions of *ACC1*, fatty acid synthase (*FAS)*, stearoyl-CoA desaturase 1 (*SCD1)*, carnitine palmityl transferase-1 (*CPT-1*), and *SREBP-1* mRNA were detected by qRT-PCR.

**Results:**

After administering YiQi for three weeks, the levels of fast blood glucose, fasting insulin, TC, TG, LDL, ALT, AST, and ALP were significantly decreased, while HDL significantly increased compared with the model group. YQ could obviously attenuate pancreatic damage and improve islet *α-* and *ß*-cell survival compared with the model group. Furthermore, YQ could attenuate hepatic damage caused by lipid accumulation, decrease the content of lipid, and increase the hepatic glycogen content, compared with the model group. In addition, YQ remarkably elevated the proteins expression of p-PI3K, p-AKT, and GLUT4 in pancreas islet and elevated the proteins expression of p-PI3K, p-AKT, GLUT4, p-AMPK, SREBP1, and PPAR*α* and inhibited the expression of p-ACC1 in liver. Besides, YQ reduced the relative expression of *ACC1*, *FAS, SERBP-1c*, and *SCD* mRNA along with the decreased production of *CPT-1* mRNA.

**Conclusions:**

YQ could attenuate type 2 diabetes mellitus by improving islet *α-* and *ß*-cells via IRS-2/AKT/GLUT4 pathway and nonalcoholic fatty liver by ameliorating lipid accumulation via AMPK/PPAR*α*/SREBP1/ACC1 pathway.

## 1. Background

Type 2 diabetes mellitus (T2DM) is a lifelong metabolic disease characterized by islet *ß*-cell dysfunction and peripheral insulin resistance resulting in hyperglycemia and hyperinsulinemia [1]. Nonalcoholic fatty liver disease (NAFLD) is a fat-associated liver damage caused by the ectopic fat accumulation in liver cells which affects hepatic structure finally leading to fibrosis and cirrhosis under uncontrolled condition [2]. 70–90% of patients with T2DM developed NAFLD, which makes it a public health problem. T2DM coexists with NAFLD with often leads to a poor glycemic control and sever complications [3–5]. Although the therapeutic strategies of T2DM had changed markedly in decade years, the optimal strategy for T2DM complicated with NAFLD remains unclear [6]. And the mainstay of T2DM complicated with NAFLD treatment is currently to reduce modifiable risk to achieve good glycemic control, such as glucagon-like peptide-1, but the adverse reaction concerns limited the use of them [7]. Therefore, it is important to explore new method for treating T2DM in patients with NAFLD.

Traditional Chinese medicines (TCM) have been used to treat diabetes and its complications for more than two thousand years in China [8]. More and more pieces of evidence have been provided for their efficacy and safety because of hypoglycemic properties, multitargets effects, and less side effects [9, 10]. YiQi YangYin Decoction (YQ) is a modern Chinese formula prescribed to treat T2DM with NAFLD based on the theory of the traditional Chinese medicine—Qi deficiency and Yin deficiency—and has been used for years at the First Affiliated Hospital of Heilongjiang University of Chinese Medicine. YQ is composed of nine traditional Chinese edible and medicinal herbs including Radix Astragali, Chinese yam, Radix ginseng, *Polygonatum odoratum*, *Cornus officinalis*, Rhizoma polygonati, Radix Puerariae, Crataegi Fructus, and fermented soya beans. Some of them including Radix Astragali, Chinese yam, American ginseng, *Polygonatum odoratum*, *Cornus officinalis*, and Rhizoma polygonati are most frequently prescribed herbs contained in antidiabetic Chinese proprietary medicines approved by the National Medical Products Administration of China (NMPA) [11]. Radix Puerariae, Crataegi Fructus, Radix ginseng, and Radix Astragali are most frequently prescribed herbs contained in lipid-lowing Chinese proprietary medicines approved by NMPA [12]. Moreover, diet formula is composed of several herbs and some bioactive component contained in eight herbs which have been reported to posse antihyperglycemic and hypolipidemic activity [13–15]. However, its mechanism for treating T2DM with NAFLD was still unclear.

Therefore, the present study aimed to evaluate pharmacological actions of YQ and to explore the possible mechanism in antihyperglycemic and hypolipidemic effects in T2DM rats.

## 2. Methods

### 2.1. YQ Decoction

The YQ prescription was prepared at the First Affiliated Hospital of Heilongjiang University of Chinese Medicine (Harbin, China): 20 g of Radix Astragali (*Astragalus propinquus* Schischkin), 20 g of Chinese yam (*Dioscorea opposita* Thunb.), 5 g of Radix ginseng (*Panax ginseng* C. A. Mey.), 10 g of *Polygonatum odoratum* (*Polygonatum odoratum* (Mill.) Druce), 10 g of *Cornus officinalis* (*Cornus officinalis* Sieb. et Zucc.), 10 g of Rhizoma polygonati (*Polygonatum sibiricum* Red.), 5 g of Radix Puerariae (*Pueraria lobata* (Willd.) Ohwi), 10 g of Crataegi Fructus (*Crataegus pinnatifida* Bunge), and 10 g of fermented soya beans were soaked with 10 times the amount of distilled water for 2 h and boiled for 1 h for 2 times. The extract was evaporated and concentrated under reduced pressure to a concentration of 0.9 g/ml (high dose) and then diluted with distilled water to 0.45 g/ml (middle dose) and 0.225 g/ml (low dose). The dose of metformin tablet used in study was 0.01 g/ml [16].

### 2.2. Chemistry Analysis of YQ by UPLC-QDa

The following conditions were used: chromatographic column: Thermo Hypersil GOLD column (100 mm × 2.1 mm, 1.9 *μ*m) (Thermo Scientific TM, Waltham, MA, USA); mobile phase: acetonitrile (A) −0.1% formic acid aqueous solution (B), gradient elution (0∼3 min, 10%–15%A; 3∼4 min, 15%∼15%A; 4∼7 min, 15%∼20%A; 7∼9 min, 20%∼35%A; 9∼15 min, 35%∼100%; 15∼16 min, 100%∼10%A); injection volume: 2 L; detection wavelength: 260 nm; column temperature: 40°C; flow rate: 0.3 ml/min; detector: Waters QDA mass spectrometer detector; ion source: electrospray ion source (ESI); scanning mode: select positive and negative ion monitoring mode and multiple reaction scanning mode; sampling rate: 8 points/s; scanning quality range: 100∼1120 Da.

### 2.3. Experimental Animals

Male SD rats (180–205 g) of specific pathogen-free grade were purchased from the Center of Laboratory Animal, Harbin Medical University (number: SCXK (Hei) 2015–0003 (Harbin, China)). All rats were housed at 22.0 ± 2.5°C and humidity 60 ± 5% on a 12 h light/dark cycle under specific pathogen-free animals' room, with free access to sterile food and water. All experiments in this study were approved by the Animal Ethics Committee of Heilongjiang University of Chinese Medicine (No. GLWJ-ZD-019).

### 2.4. Preparation of T2DM Model

Fifty-five rats were allowed to acclimate for at least three days. After fasting 8 h, levels of blood glucose (FBG) of rats were measured by a blood glucose meter (ACCU-CHEK, Roche, Germany). Then, rats were divided into the control group (normal diet, *n* = 6) and HFD group (high-fat diet, *n* = 49). High-fat diet consisted of 67% normal diet, 10% lard, 20% sucrose, 2.5% cholesterol, and 0.5% sodium cholate (Beijing Keao Xieli Feed Co., Ltd, China). With feeding with high-fat diet for 4 weeks, rats in HFD group were intraperitoneally injected with freshly prepared streptozotocin (STZ) at a dose of 35 mg/kg (dissolved in 0.1 mol/L sodium citrate buffer pH 4.5) after fasting 16 h. Three days and seven days after STZ treatment, the levels of FBG in HFD group greater than 11.1 mmol/L were considered for diabetic rats.

### 2.5. Animal Treatment

**Figure 1:**
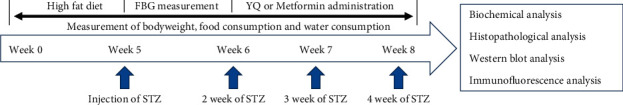
Experimental design.

Forty-six diabetic rats were randomly divided into five groups including model group (*n* = 10), positive control group (0.2 g metformin tablet/kg body weight per day, *n* = 9), YQ high-dose group (14 g/kg body weight per day, *n* = 9), YQ middle-dose group (7 g/kg body weight per day, *n* = 9), and YQ low-dose group (3.5 g/kg body weight per day, *n* = 9). All rats were orally given corresponding ingredients for three weeks. During the experimental period, bodyweight was measured every week. Food consumption and water consumption were measured during the experimental period in all groups. The rats in each group received 1300 mL of tap water and 500 g of high-fat diet food at 3 pm every day. After 24 h, the volume of leftover water and quantity of leftover food were measured. Weekly average food consumption and water consumption were calculated. The experimental flow graph is shown in Figure 1.

### 2.6. Measurement of Fasting Blood Glucose (FBS) and Fasting Insulin (Fins)

After fasting 8 h, blood samples were collected from the tip of the tail. The level of fast blood glucose was measured by a blood glucose meter (ACCU-CHEK, Roche, Germany). The level of fasting insulin was evaluated by using an Elisa kit (Chenglin Biotechnology Co., Ltd., Beijing, China).

### 2.7. Tissue Preparation

At the end of experiment, all rats were anaesthetized by 3% sodium pentobarbital at a dose of 0.1 mL/100 g body weight. The blood samples were collected from abdominal aorta into vacuum blood collection tubes with heparin (35 IU/ml) for blood viscosity. Serum was obtained by centrifugation at 3000x rpm for 10 min and stored at −80°C for biochemical analysis. The tissues including liver and pancreas were separated and weighted. One part of tissues was immediately frozen in liquid nitrogen for western blot analysis. Another part was fixed in 4% polyformaldehyde for histopathological analysis.

### 2.8. Biochemical Analysis

Serum samples were used to determine the levels of total cholesterol (TC, lot 187836), triglyceride (TG, lot 187831), high-density lipoprotein (HDL, lot 180721), low-density lipoprotein (LDL, lot 191301), alanine aminotransferase (ALT, lot 182681), aspartate aminotransferase (AST, 181361), alkaline phosphatase (ALP, lot 181511), and total protein (TP, 181511) according to the manufacturer instructions (Institute of Biological Engineering of Nanjing Jiancheng, Nanjing, China).

### 2.9. Histopathological Analysis

The liver and pancreas tissues were fixed in 4% polyformaldehyde for 24 h. Then, these tissues were embedded in paraffin and cut into 5 *μ*m slices. After dewaxing, slices were stained with hematoxylin eosin (HE) for histopathological examinations. The Periodic Acid-Schiff (PAS) staining was used to detect the content of glycogen in liver. Oil red O staining was used to detect fat deposition in liver.

### 2.10. Western Blot Analysis

The liver and pancreas tissues (100 mg) were homogenized in RIPA lysis buffer (1 ml) on ice for 30 min. The lysate was centrifuged at 12000 rpm at 4°C for 15 min. The supernatant was collected and protein concentration was determined by using a BCA Protein Assay Kit. A total of 100 *μ*g of protein was separated on 6%–12% separating gel and transferred onto BioTrace nitrocellulose membranes (Pore Size 0.2 *μ*m). The membranes were blocked with 5% block milk or BSA for 1 h at room temperature and then incubated with primary antibodies for IRS-2 (1 : 3000, lot DF7534), P-PI3K (1 : 3000, lot AF4371), PI3K (1 : 3000, lot AF6241), AKT (1 : 5000, lot WL0003b), P-AKT (1 : 5000, lot WLp1001a), GLUT4 (1 : 3000, DF7510), P-AMPK (1 : 3000, lot AF3423), AMPK (1 : 5000, LOT WL02254), SREBP1 (1 : 5000, lot wl02093), P-ACC1 (1 : 3000, lot AF3421), ACC1 (1 : 3000, lot AF6241), PPAR*α* (1 : 5000, lot WL00978), and *ß*-actin (1 : 5000, lot WL01845) overnight at 4°C. After washing the membranes with TBST, membranes were incubated with HRP-goat anti-rat or HRP-rabbits anti-rat antibodies (1 : 5000, lot WL02093) for 2 h at room temperature. 500 *μ*L working solution of the Clarify Western ECL Substrate was added to the surface of membrane to ensure that the whole membrane was covered with working solution for 3 minutes under no light condition. The protein bands were detected by using a ChemiDoc XRS^+^ system (Bio-Rad, Hercules, USA) and analyzed by using ImageJ 1.52 v (National Institutes of Health, Bethesda, MD, USA).

### 2.11. Quantitative Real-Time PCR (qRT-PCR)

The total RNA was extracted from the liver and using TRIpure regent (RP1001, BioTeke). The cDNA was synthesized from RNA by a reverse transcription reagent kit (PR6502, BioTeke). The expressions of these genes (shown in Table 1) were determined by qRT-PCR and using SYBR Green PCR kit (SY1020, Solarbio), which were performed on Real-Time PCR System (ExicyclerTM 96, BIONEER, Korea). *ß*-Actin was used as a reference gene and comparison in expression between groups was made using the 2^−ΔΔCT^ method.

### 2.12. Immunofluorescence Analysis

Fixed pancreas tissues were embedded in paraffin and cut into slices at 5 *μ*m thickness. The slices were soaked in xylene and ethanol for rehydrating. Then, slices were incubated with 10% citrate buffer (pH 8.0) for antigen retrieval in a microwave oven for 10 min. After blocking with normal goat serum for 15 min at room temperature, the slices were incubated with the primary antibodies for insulin (1 : 3000) and glucagon (1 : 3000) at 4°C overnight. After incubating with Cy3-goat anti-rabbit secondary antibody for 60 min at room temperature, DAPI dye was used to label the cell nucleus. The images were captured with a fluorescence microscopy (IX73, Olympus, Japan). The areas of islet *α*-cell and islet *ß*-cell were measured by using ImageJ 1.52 v (National Institutes of Health, Bethesda, MD, USA).

### 2.13. Statistical Analysis

All data are expressed as the mean value±standard deviation. The data are analyzed using SPSS 16.0 software according to the following methods: One-sample Kolmogorov-Smirnov test was used for analyzing the distribution of data. Homogeneity of variance was examined by Bartlett's test. One-way analysis of variance (ANOVA) was applied for homogeneous data and Dunnett's test was used for multiple comparisons. Dunnett's T3 test was used for heterogeneous data. The value of *P* of less than 0.05 (*P* < 0.05) was considered statistically significant.

## 3. Results

### 3.1. Chemistry Analysis of YQ

**Figure 2:**
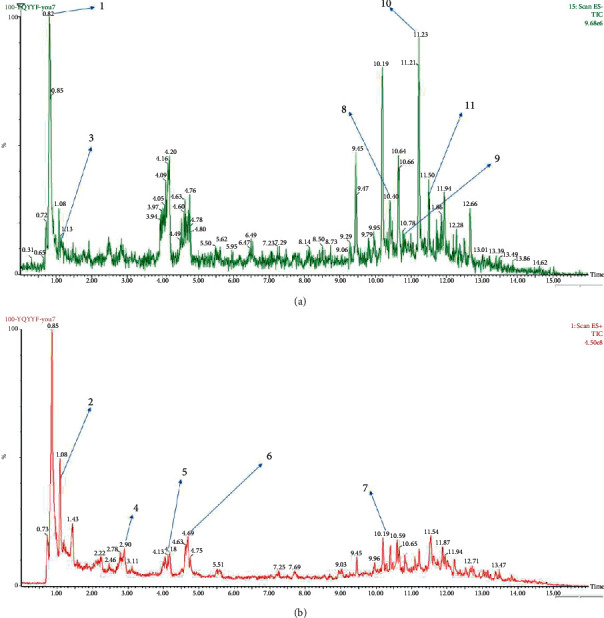
The chemistry analysis of YQ by UPLC-QDa. (a) Negative ion monitoring mode. (b) Positive ion monitoring mode.

As is shown in Figure 2 and Table 2, YQ was analyzed by UPLC and the results showed that citric acid, adenine, gallic acid, morroniside, puerarin, calycosin-7-glucoside, daidzein, ginsenoside Re, quercetin, genistein, and ginsenoside Rb1 were the main compounds of YQ.

### 3.2. Effect of YQ on Body Weight, Food Intake, and Water Consumption

**Figure 3:**
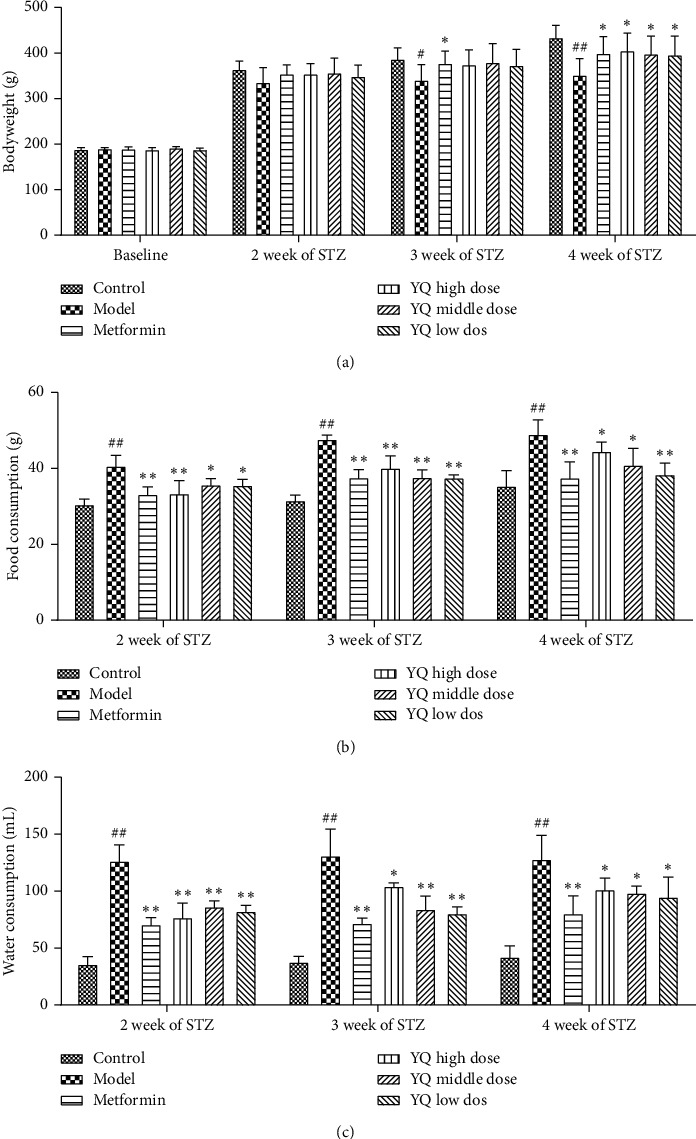
Effect of YQ on body weight, food intake, and water consumption. (a) The changes of bodyweights during experimental period. (b) The food consumption among groups during experimental period. (c) The water consumption among groups during experimental period. The results are shown as means ± SEM (*n* = 9-10). ^#^*P* < 0.05, ^##^*P* < 0.01 compared with the control group; ^*∗*^*P* < 0.05, ^*∗∗*^*P* < 0.01 compared with the model group.

As shown in Figure 3(a), before administration, there was no difference in the body weight among all groups. After administration for three weeks, the body weight in the model group significantly decreased (*P* < 0.01) compared with the control group. Three doses of YQ significantly increased (*P* < 0.05) the bodyweights of diabetic rats compared with the model group.

As shown in Figures 3(b) and 3(c), compared with the control group, the food consumption and water consumption in mode group were significantly increased (*P* < 0.01). During administration period, three doses of YQ significantly decreased (*P* < 0.05, *P* < 0.01) the consumption of food and water of diabetic rats compared with the model group.

### 3.3. Effect of YQ on Fasting Blood Glucose Level and Fasting Insulin Level

**Figure 4:**
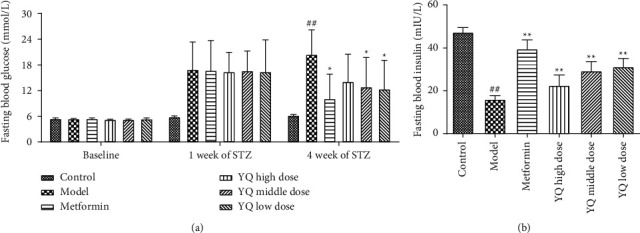
Effect of YQ on fasting blood glucose level and fasting insulin level. The results are shown as means ± SEM (*n* = 9-10). ^##^*P* < 0.01 compared with the control group; ^*∗*^*P* < 0.05, ^*∗∗*^*P* < 0.01 compared with the model group.

As shown in Figure 4, during experimental period, the level of fasting blood glucose (FBG) in the model group obviously increased from 5.40 ± 0.29 mmol/l to 20.49 ± 5.91 mmol/l. After treatment for three weeks, the level of FBG in YQ low-dose and YQ middle-dose groups obviously decreased (*P* < 0.05) compared with the model group. Besides, the level of fasting serum insulin (Fins) in the model group was significantly lower than that in the control group. After administering YQ for three weeks, the level of Fins in YQ low dose and YQ middle-dose groups was significantly increased (*P* < 0.01) compared with the model group.

### 3.4. Effect of YQ on the Levels of Lipids in Serum

**Figure 5:**
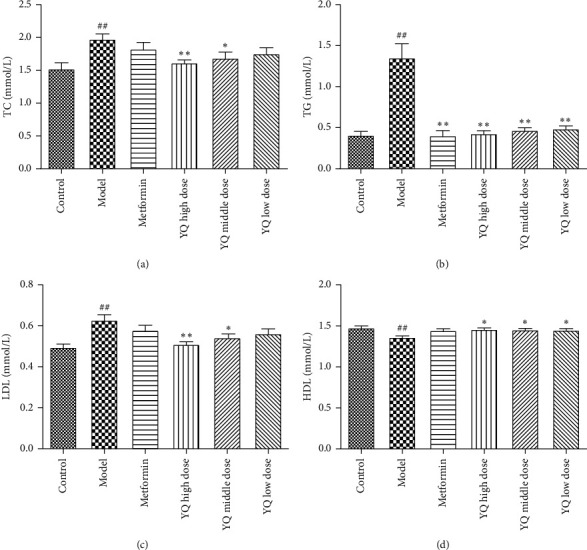
Effect of YQ on the levels of lipids in serum. (a) The level of TC in serum. (b) The level of TG in serum. (c) The level of LDL in serum. (d) The level of HDL in serum; the results are shown as means ± SEM (*n* = 9-10). ^##^*P* < 0.01 compared with the control group; ^*∗*^*P* < 0.05, ^*∗∗*^*P* < 0.01 compared with the model group.

As shown in Figure 5, compared with the control group, the levels of TC, TG, and LDL in serum were significantly elevated (*P* < 0.01); HDL decreased (*P* < 0.01) in the model group. After treatment for three weeks, YQ could obviously reduce the levels of TC, TG, and LDL (*P* < 0.05 or *P* < 0.01) and elevate HDL (*P* < 0.05) in serum compared with the model group. Metformin significantly reduced the level of TG (*P* < 0.01) but has no significant effect on TC, LDL, and HDL in serum.

### 3.5. Effect of YQ on Histopathological Changes of Pancreas Islet and Liver

**Figure 6:**
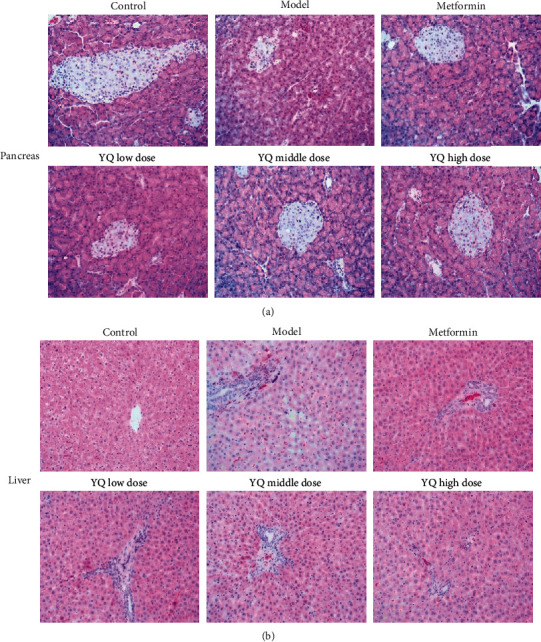
Effect of YQ on histopathological changes of pancreas islet and liver. At the end of experiment, tissues including pancreas islet and liver were removed in each group for hematoxylin and eosin (HE) dye. The histopathological changes were observed and pictured under optical microscope (200x).

As shown in Figure 6(a), compared with the control group, the pancreatic islet cells in the endocrine area showed irregular shape, the boundary between islet cells and acinus was blurred, the islet cells were atrophic and distributed unevenly, and more local nuclear necrosis and lysis were observed in model group. Exocrine acini were swollen and the boundary among acini was blurred. Compared with the model group, the pancreatic islet cells in YQ high-dose group were distributed in a mass, with clear and intact edges, and intact nuclei and exocrine acinus were irregular polygonal, neatly arranged, and the boundary among acinus was clearly visible. In the middle-dose groups, the above pathological conditions were also improved. As shown in Figure 6(b), compared with the control group, hepatocyte swelling, steatosis with lots of intercellular vacuoles, and infiltration of inflammatory cells were observed in model group. Compared with the model group, the degree of hepatocyte steatosis and the number of intercellular vacuoles in YQ high- and middle-dose groups were both significantly reduced, and hepatocytes were normal in size, orderly, and clear in structure.

### 3.6. Effect of EAF on Liver Index and Liver Enzyme Activities

**Figure 7:**
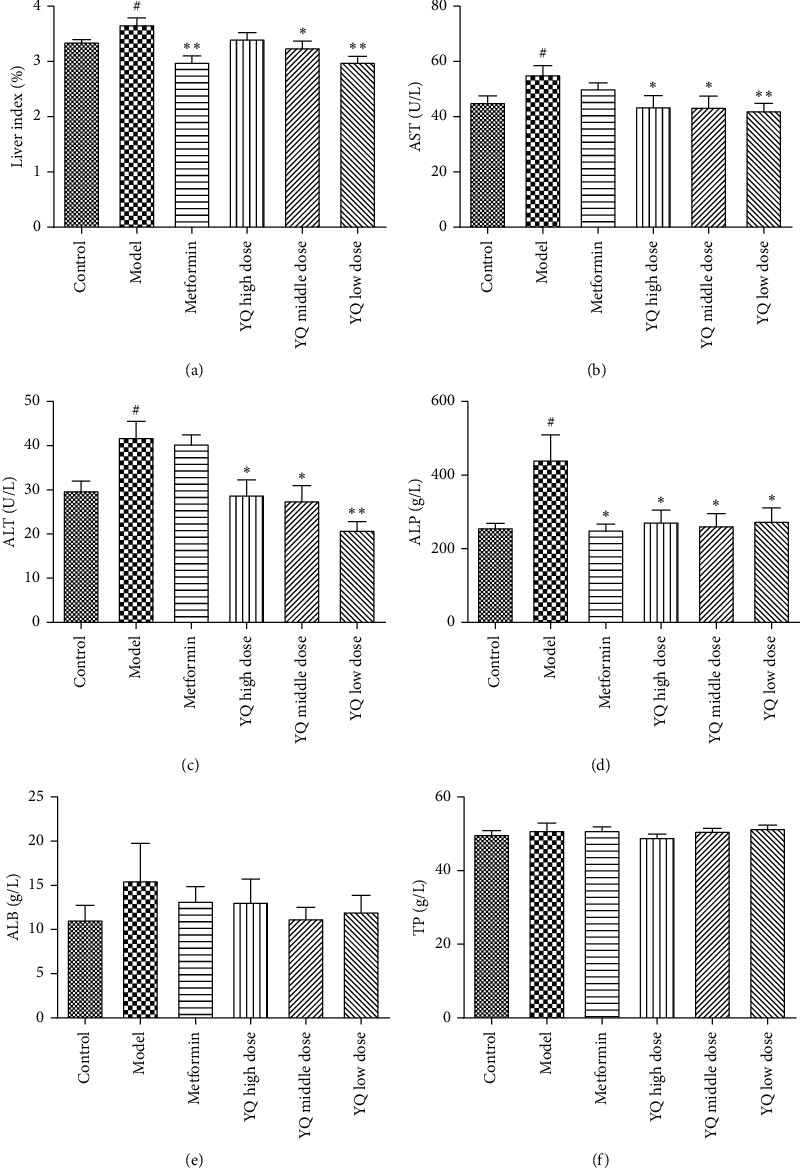
Effect of YQ on liver index and liver enzyme activities. (a) The liver index in each group. (b) The level of AST in serum. (c) The level of ALT in serum. (d) The level of ALP in serum. (e) The level of ALB in serum. (f) The level of TP in serum. Results are shown as means ± SEM (*n* = 9-10). ^##^*P* < 0.01 compared with the control group; ^*∗*^*P* < 0.05, ^*∗∗*^*P* < 0.01 compared with the model group.

As shown in Figure 7(a), compared with the control group, the liver index was significantly increased (*P* < 0.01) in the model group. After treatment for three weeks, the liver index in YQ low dose and YQ middle-dose groups obviously decreased (*P* < 0.05 or *P* < 0.01) compared with the model group. As shown in Figures 7(b)–7(d), compared with the control group, the content of ALT, AST, and ALP in YQ dose groups obviously decreased (*P* < 0.05 or *P* < 0.01) compared with the model group. However, the content of TP and ALB was not obviously changed (*P* > 0.05).

### 3.7. Effect of YQ on the Area of Islet *α-* and Islet *ß*-Cell

**Figure 8:**
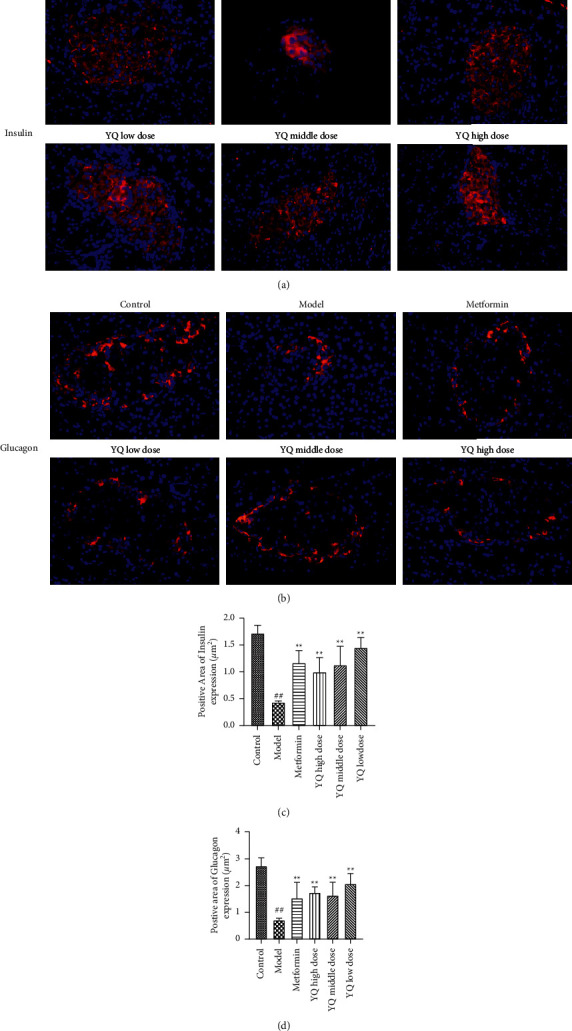
Effect of YQ on the area of islet *α-* and islet *ß*-cell. At the end of experiment, pancreas islet was removed in each group for immunofluorescence analysis. (a) Insulin-positive cells. (b) Glucagon-positive cells. The fluorescent areas of insulin-positive and glucagon-positive cells were observed and pictured under fluorescence microscopy (200x). (c, d) The areas of positive cells were measured by using ImageJ 1.52 v software. The results are shown as means ± SEM (*n* = 9-10). ^##^*P* < 0.01 compared with the control group; ^*∗∗*^*P* < 0.01 compared with the model group.

As shown in Figure 8, compared with the control group, the positive area with red fluorescence of islet *α-* and islet *ß*-cell was significantly decreased (*P* < 0.01) in the model group. After treatment for three weeks, islet *α-* and islet *ß*-cell in YQ dose groups obviously decreased (*P* < 0.05, *P* < 0.01) compared with the model group.

### 3.8. Effect of YQ on Glycogen Synthesis and Steatosis in Liver

**Figure 9:**
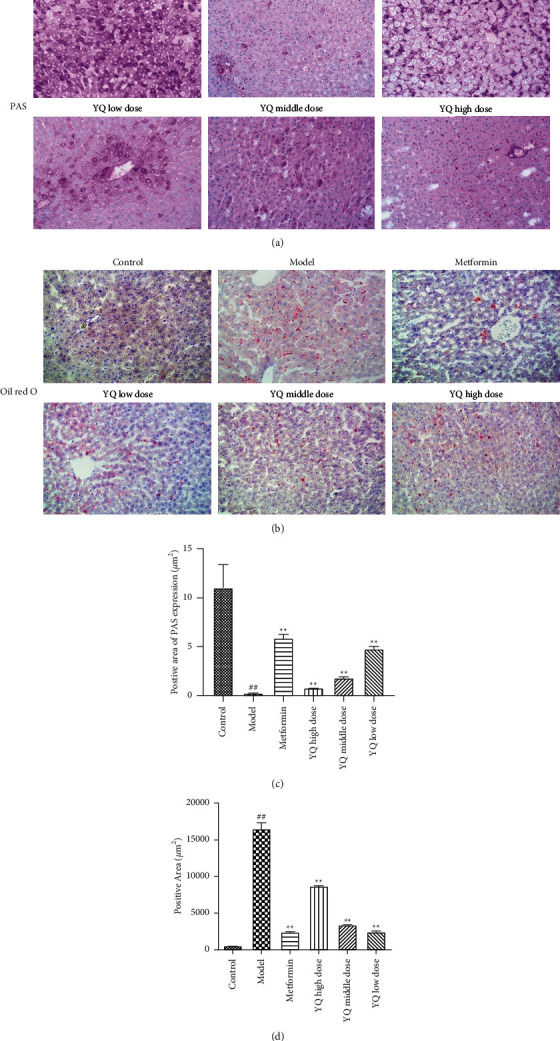
Effect of YQ on glycogen synthesis and steatosis in liver. At the end of experiment, liver tissue was removed in each group. (a) PAS staining. “⟶” pointed at the content of glycogen. (b) Oil red O staining. “△” pointed at lipid. The positive cells were observed and pictured under optical microscope (200x). (c, d) The area of positive cells was measured by using ImageJ 1.52 v software. The results are shown as means ± SEM (*n* = 9–10). ^##^*P* < 0.01 compared with the control group; ^*∗∗*^*P* < 0.01 compared with the model group.

As shown in Figure 9(a), compared with control group, the content of glycogen was significantly decreased (*P* < 0.01) in model group. After treatment for three weeks, the content of glycogen in YQ low-dose and YQ middle-dose groups obviously increased (*P* < 0.05, *P* < 0.01) compared with the model group.

As shown in Figure 9(b), compared with control group, the content of lipid was significantly increased (*P* < 0.01) in model group. After treatment for three weeks, the content of glycogen in all three YQ dose groups obviously decreased (*P* < 0.05 or *P* < 0.01) compared with the model group.

### 3.9. Effect of YQ on Lipid Metabolism-Related Proteins in Liver

**Figure 10:**
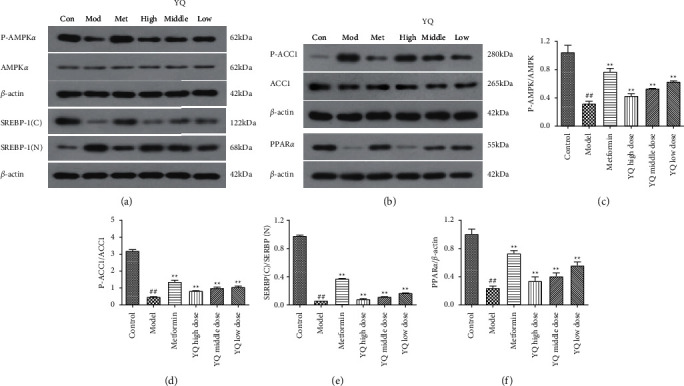
Effect of YQ on lipid metabolism-related proteins in liver. The expressions levels of P-AMPK*α*, AMPK*α*, PPAR*α*, P-ACC1, ACC1, SERBP-1 (cytoplasm), and SERBP-1 (nucleus) were assessed by western blot analysis. The results are shown as means ± SEM (*n* = 9–10). ^##^*P* < 0.01 compared with the control group; ^*∗∗*^*P* < 0.01 compared with the model group.

As shown in Figures 10(a)–10(f), compared with the control group, the expression levels of P-AMPK*α*, SERBP-1 (cytoplasm), PPAR*α*, and P-ACC1 were significantly decreased (*P* < 0.01), while the expression levels of SERBP-1 (nucleus) significantly improved in model group. After treatment for three weeks, the expression levels of P-AMPK*α*, SERBP-1 (cytoplasm), PPAR*α*, and P-ACC1 in all three YQ dose groups were significantly increased, while the expression levels of SERBP-1 (nucleus) significantly (*P* < 0.01) reduced compared with the model group.

### 3.10. Effect of YQ on Insulin Secretion-Related Proteins in Pancreas Islet

**Figure 11:**
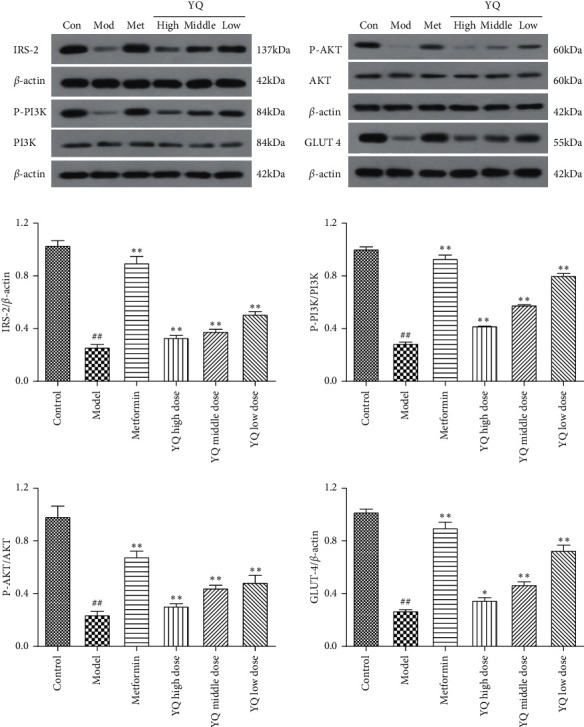
Effect of YQ on insulin secretion-related proteins in pancreas islet. The expressions levels of IRS-2, P-PI3K, PI3K, P-Akt, Akt, and GLUT4 were assessed by western blot analysis. The results are shown as means ± SEM (*n* = 9–10). ^##^*P* < 0.01 compared with the control group; ^*∗*^*P* < 0.05, ^*∗∗*^*P* < 0.01 compared with the model group.

As shown in Figure 11, compared with the control group, the expression levels of IRS-2, P-PI3K, P-AKT, and GLUT4 were significantly decreased (*P* < 0.01). After treatment for three weeks, the expression levels of IRS-2, P-PI3K, P-AKT, and GLUT4 in all three YQ dose groups were significantly increased (*P* < 0.05 or *P* < 0.01) compared with the model group.

### 3.11. Effect of YQ on the Expression of Related Fatty Targets Genes

**Figure 12:**
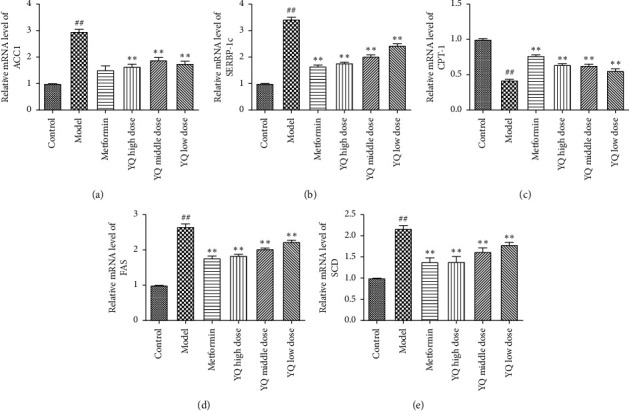
Effects of YQ on the expression of target genes involved in fatty acid synthesis pathway, lipolysis pathway, or fat transport. The expressions of *ACC*1 (a), *SERBP*-1c (b), *CPT-*1 (c), *FAS* (d), and *SCD* (e) were detected by qRT-PCR. The results are shown as means ± SEM (*n* = 9-10). ^##^*P* < 0.01 compared with the control group; ^*∗*^*P* < 0.05, ^*∗∗*^*P* < 0.01 compared with the model group.

In order to further explore the effect of YQ on the fat and lipolysis production, the related targets gene levels were determined. Compared with the control group, the relative expressions of *ACC*1, *FAS, SERBP-1c*, and *SCD* were increased along with the decreased production of *CPT-*1 in model group (*P* < 0.01). And as expected, the expressions of these genes were all reserved after the treatment with YQ (*P* < 0.01, Figure 12).

## 4. Discussion

High-fat diet feeding combined with a single or multiple intraperitoneal injections of low dose STZ has been known to successfully establish experimental type 2 diabetic animal model [17]. Hyperinsulinemia is induced by feeding with high-fat diet at the early stage of development of type 2 diabetes. However, at later stage, the level of insulin is relatively reduced because of the destruction of islet *ß*-cell caused by STZ [18, 19]. In our study, the levels of serum insulin were relatively decreased accompanied with high blood glucose in model group compared with the control group, which is consistent with the results of previous study [20]. The results demonstrated that, compared with the model group, YQ treatment significantly restored the levels of insulin and decreased the levels of FBG. Besides, YQ treatment also significantly increased the body weight and decreased the consumption of food and water of diabetic rats. Progressive decline of islet *ß*-cell function and partial loss of islet *ß*-cell mass were the pathological features of type 2 diabetes [21–23]. Pathological analysis showed that the islet *ß*-cells were atrophic and were distributed unevenly, and local nuclear effects were necrosis and lysis in model group, which were obviously improved by YQ treatment. Above findings indicate YQ can obviously improve clinical features of type 2 diabetes by improving pathological damage of islet cells.

Nonalcoholic fatty liver disease (NAFLD) is the most common liver disease and is highly associated with obesity and diabetes mellitus, which affect up to 25% of the general population globally [24]. NAFLD and T2DM are often coexisting diseases that synergistically increase the incidence of diabetic complications and the risk of liver fibrosis, cirrhosis, liver cancer, and even cardiovascular events [7, 25]. NAFLD can be determined when lipid droplets present in more than 5% of the hepatocytes in liver biopsy [26]. NAFLD is also characterized by elevated levels of LDL-C and TG and by decreased HDL-C concentrations [27]. Elevated liver enzymes are only found in approximately 20% of patients with NAFLD, although liver enzymes are a clinical manifestation of liver function [28]. In present study, the hepatocytes were steatosis obviously in the model group, and YQ reduced the degree of hepatocyte steatosis and the number of intercellular vacuoles as expected. The serum levels of LDL-C, TG, ALT, AST, and ALP were significantly increased, while HDL-C concentrations significantly decreased in model group. The changes mentioned above were significantly ameliorated by YQ treatment, suggesting the beneficial effects of ameliorating lipid accumulation and protecting the liver damage.

AMP-activated protein kinase (AMPK) is a metabolism monitor that plays an important role in energy balance and glucose and lipid homeostasis in diabetes mellitus [29]. Activated AMPK*α* promotes phosphorylation of acetyl-CoA carboxylase (ACC) and inhibits its activation. The inactivation of ACC reduces the synthesis of Malonyl-CoA, which leads to the disinhibition of CPT-1, finally increasing *ß*-oxidation of fatty acid and inhibiting lipid synthesis [30, 31]. In addition, activated AMPK*α* can repress fat accumulation and hepatic steatosis by regulating downstream gene PPAR*α* and SREBP-1, known as two important transcription factors in lipid metabolism [32, 33]. PPAR*α* is mainly responsible for the transport of fatty acid and advancement of beta-oxidation in mitochondria [34]. Sterol regulatory element-binding protein 1c (SREBP1c) activates the expression of genes involved in fatty acid and cholesterol synthesis in liver [35]. In present study, YQ treatment remarkably increased the expression of P-AMPK*α*, P-ACC1, PPAR*α*, and SERBP1 (cytoplasm). Therefore, above results indicated that YQ can increase lipid metabolism by activating the AMPK*α*/ACC1/PPAR*α*/SERBP1 pathway.

Insulin receptor substrate (IRS) proteins act as adaptor molecule responding to extracellular stimulants by coupling transmembrane receptors including insulin receptor and insulin-like growth factor 1 (IGF-1) receptor and regulate diverse biological processes such as glucose metabolism, cell growth, and cell survival [36]. The IRS-2 protein coordinates insulin/IGF-1 signaling transduction pathways, which is critical for islet *ß*-cell growth and function, and its deficiency will lead to a decrease in islet *ß*-cells mass [37]. Phosphatidylinositol 3-kinase (PI3K)/Akt (protein kinase B) cascade is one of the two major downstream signaling pathways activated by IRS-2 protein [38]. After phosphorylation by activated insulin/IGF-1 receptor on tyrosine residues, phosphorylated IRS-2 binds to the regulatory subunit *p*85*α* of PI3K, thereby activating the PI3K enzyme. Activated PI3K converts PI-4,5-P_2_ to PI-3,4,5-P_3_ that recruits phosphoinositide-dependent protein kinase-1 (PDK1) and AKT, leading to phosphorylation of Akt at Thr306 and Ser473 by PDK1 in the plasma membrane [39, 40]. Activated Akt can regulate the size, number, and intracellular gene transcription of pancreatic beta cells through different downstream substrates. The immunofluorescence analysis showed that the mass of islet *ß*-cells was obviously smaller in model groups than that of normal control group. However, it was obviously improved by YQ treatment. The results of WB showed that the expression levels of IRS-2, P-PI3K, P-AKT, and GLUT4 proteins were significantly elevated after YQ treatment compared with the model group. These results indicated that YQ could improve the growth of islet *ß*-cells involved in the IRS-2/PI3K/AKT signaling pathway.

## 5. Conclusion

**Figure 13:**
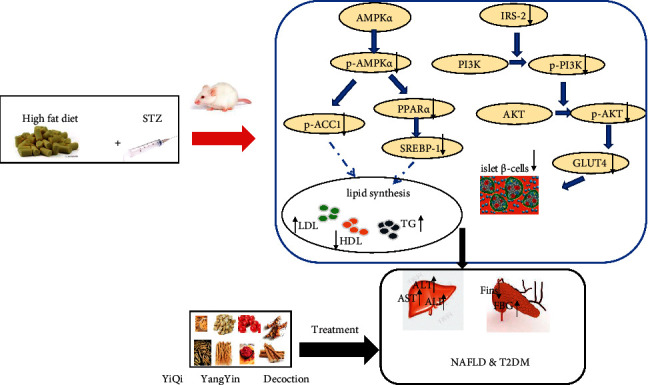
Mechanism of YiQi YangYin Decoction in the treatment of T2DM and NAFLD.

YQ could attenuate type 2 diabetes mellitus by improving *ß*-cell via IRS-2/AKT/GLUT4 pathway and NAFLD by ameliorating lipid accumulation via AMPK*α*/ACC1/PPAR*α*/SREBP1 pathway (Figure 13).

## Figures and Tables

**Table 1:** PCR primers.

Gene	Sequence
ACC1	F: 5′-TGAGGAGGACCGCATTTATC-3′
	R: 5′-TGAGGAGGACCGCATTTATC-3′

FAS	F: 5′-TGAGGAGGACCGCATTTATC-3′
	R: 5′-TGCTGTAGCCCAGAAGAG-3′

SCD1	F: 5′-ATCGCCCCTACGACAAGAAC-3′
	R: 5′-AGGAACTCAGAAGCCCAGAAC-3′

CPT-1	F: 5′-ACGAGCCGATTGGGCTAAA-3′
	R: 5′-ACCAACGATCGTGAGCCTTT-3′

SREBP-1	F: 5′-GCTCACAAAAGCAAATCACT-3′
	R: 5′-GCGTTTCTACCACTTCAGG-3′

*β*-Actin	F: 5′-GGAGATTACTGCCCTGGCTCCTAGC-3′
	R: 5′-GGCCGGACTCATCGTACTCCTGCTT-3′

**Table 2:** Compounds of YQ.

Number	Compound	Chemical structure	Formula
1	Citric acid	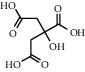	C_6_H_8_O_7_
2	Adenine	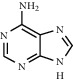	C_5_H_5_N_5_
3	Gallic acid	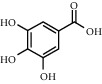	C_7_H_6_O_5_
4	Morroniside	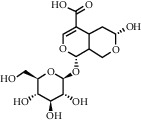	C_17_H_26_O_11_
5	Puerarin	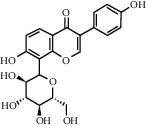	C_21_H_20_O_9_
6	Calycosin-7-glucoside	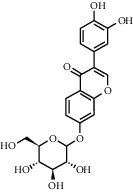	C_22_H_22_O_10_
7	Daidzein	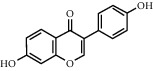	C_15_H_10_O_4_
8	Ginsenoside Re	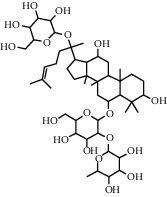	C_48_H_82_O_18_
9	Quercetin	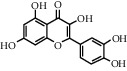	C_15_H_10_O_7_
10	Genistein	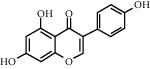	C_15_H_10_O_5_
11	Ginsenoside Rb1	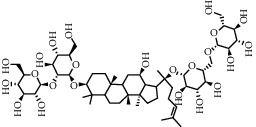	C_54_H_92_O_23_

## Data Availability

The datasets used and/or analyzed during the current study are available from the corresponding author on reasonable request.

## Abbreviations

YQ: YiQi YangYin decoction
T2DM: Type 2 diabetes mellitus
TC: Total cholesterol
TG: Triglyceride
HDL: High-density lipoprotein
LDL: Low-density lipoprotein
ALT: Alanine aminotransferase
AST: Aspartate aminotransferase
ALP: Alkaline phosphatase
PAS: Periodic acid-Schiff
IRS-2: Insulin receptor substrate 2
PI3K *p*85*α*: Phosphatidylinositol 3-kinase-associated *p*85alpha
GLUT4: Glucose transporter 2
AMPK*α*: AMP-activated protein kinase alpha
SREBP1c: Sterol regulatory element-binding protein 1c
ACC1: Acetyl-CoA carboxylase 1
PPAR*α*: Peroxisome proliferator‐activated receptor-*α*
NMPA: National Medical Products Administration of China
